# Divergent antioxidant capacity of human islet cell subsets: A potential cause of beta-cell vulnerability in diabetes and islet transplantation

**DOI:** 10.1371/journal.pone.0196570

**Published:** 2018-05-03

**Authors:** Atsushi Miki, Camillo Ricordi, Yasunaru Sakuma, Toshiyuki Yamamoto, Ryosuke Misawa, Atsuyoshi Mita, Ruth D. Molano, Nosratola D. Vaziri, Antonello Pileggi, Hirohito Ichii

**Affiliations:** 1 Cell Transplant Center, Diabetes Research Institute, University of Miami, Miami, Florida, United States of America; 2 Department of Medicine, University of California, Irvine, United States of America; 3 Department of Surgery, University of California, Irvine, United States of America; Children’s Hospital Boston, UNITED STATES

## Abstract

**Background:**

Type 1 and Type 2 diabetes mellitus (T1DM and T2DM) are caused by beta(β)-cell loss and functional impairment. Identification of mechanisms of β-cell death and therapeutic interventions to enhance β-cell survival are essential for prevention and treatment of diabetes. Oxidative stress is a common feature of both T1DM and T2DM; elevated biomarkers of oxidative stress are detected in blood, urine and tissues including pancreas of patients with DM. Islet transplantation is a promising treatment for diabetes. However, exposure to stress (chemical and mechanical) and ischemia-reperfusion during isolation and transplantation causes islet loss by generation of reactive oxygen species (ROS). Human intracellular antioxidant enzymes and related molecules are essential defenses against ROS. Antioxidant enzyme levels including superoxide dismutase (SOD), catalase, and glutathione peroxidase (GPX) have been shown to be low in islet cells. However, little is known about the expression and function of antioxidant enzymes within islet cell subsets. We evaluated the expression of the key antioxidant enzymes in β- and alpha(α)-cell and accessed effects of oxidative stress, islet isolation and transplantation on β/α-cell ratio and viability in human islets.

**Methods:**

Human pancreata from T1DM, T2DM and non-diabetic deceased donors were obtained and analyzed by confocal microscopy. Isolated islets were (I) transplanted in the renal sub-capsular space of streptozotocin-induced diabetic nude mice (*in vivo* bioassay), or (II) exposed to oxidative (H_2_O_2_) and nitrosative (NO donor) stress for 24 hrs *in vitro*. The ratio, % viability and death of β- and α-cells, and DNA damage (8OHdG) were measured.

**Results and conclusions:**

Catalase and GPX expression was much lower in β- than α-cells. The β/α-cell ratio fells significantly following islet isolation and transplantation. Exposure to oxidative stress caused a significantly lower survival and viability, with higher DNA damage in β- than α-cells. These findings identified the weakness of β-cell antioxidant capacity as a main cause of vulnerability to oxidative stress. Potential strategies to enhance β-cell antioxidant capacity might be effective in prevention/treatment of diabetes.

## Introduction

The prevailing obesity and sedentary life style has resulted in the pandemic of type 2 diabetes mellitus (T2DM), which is responsible for a great share of the health care resources worldwide. Type 1 and type 2 diabetes (T1DM and T2DM) are caused by β-cell dysfunction and accelerated death, which occur at a much greater extent in T1DM. Identification of the underlying mechanisms and development of therapeutic interventions to enhance growth and survival of β-cells are essential for prevention and treatment of diabetes.

In genetically susceptible persons, environmental factors such as viruses and toxins, trigger autoimmune destruction of β-cells which leads to T1DM usually in children and in young adults[1]. T2DM usually results from peripheral insulin resistance and β-cell dysfunction. Oxidative stress is a common feature of both T1DM and T2DM, as evidenced by numerous studies which have demonstrated accumulation of biochemical markers of oxidative stress in the blood, urine and various tissues including pancreas of patients with DM[2, 3]. Islet cell transplantation has become a promising intervention for patients with T1DM[4]. During islet isolation and following transplantation, islets are exposed to deleterious conditions such as ischemia, chemical and mechanical insults, severe ischemia-reperfusion injury promoting generation of reactive oxygen species (ROS), causing damage and significant loss of functional islet mass[5–7]. Human islet preparations used for transplantation show wide variation in β-cell content[8–10]. In addition, the fact that β-cell proportion in human islets is significantly lower than the 60–70% found in rodent islets indicates that properties of islet in human are different from that of other species[11]. Exposure to uncontained ROS for prolonged periods leads to cell injury and tissue damage[12–19].

Antioxidant enzymes and related molecules contained within host cells and tissues are the first line of defense against ROS. It has been reported that the pancreatic islet is among the least endowed tissues in terms of intrinsic antioxidant enzyme expression and activity, including superoxide dismutases (SOD-1, SOD-2), catalase, and glutathione peroxidase (Gpx). However, very little is known about the expression of antioxidant enzymes within human islet cell subset.

The present study was undertaken to investigate: (I)- β/α-cell ratio in human islets in various stress conditions where islet cells are exposed to substantial oxidative stress, and (II)- to explore the cause of the vulnerability of human β-cells against oxidative stress when compared with other islet cells, such as α-cells.

## Materials and methods

### Human pancreas and islet isolation

Human pancreata obtained from multi-organ deceased donors were utilized for research at the University of Miami’s Diabetes Research Institute (DRI) Human Cell Processing Core. The study was reviewed and approved by the University of Miami institutional review board. Our study does not involve any information including living individuals, identifiable and private information. Islet cells were isolated using a modified automated Ricordi method. Briefly, pancreatic digestion was performed using Liberase HI (1.4mg/ml; Roche, Indianapolis, IN) in the Ricordi chamber. The digested pancreatic tissues were washed with RPMI medium using centrifugation 3 times (180g, 1min, 4°C). Final collected samples were suspended with University of Wisconsin solution up to 100ml. Continuous gradient purification was performed using 1.100 and 1.077 Ficoll gradients[20]. The tissues (<25ml/run) were loaded into the COBE bag. For 5 min, they were spun (2400rpm, 4°C) and collected into 7 fractions using a refrigerated computed cell processor (Cobe2991; COBE Laboratories Inc., Lakewood, CO). Counting samples were then taken to evaluate % trap and pre-purification yield. The purity and islet yield were examined by dithizone dye[21, 22].

### Sample preparation

Before isolation, a piece of pancreas was cut and embedded in compound for cryopreservation and preserved at 80°C. For *in-vitro* examination, after isolation 2000iEQ islets were immediately collected and fixed in Bouin’s fixative solution (Sigma-Aldrich, St. Louis, MO). And islets were cultured for farmer 24 hours at 22 °C, 5% CO_2_ and for former 24 hours at 48°C, 5% CO_2_. After the culture, islets were used for following experiments.

### Assessment of islet cell subset vulnerability to oxidative stress

Islets were treated with the following compounds to simulate the effects of oxidative and nitrosative stress, as previously described[23]. Briefly, non-diabetes donor islet after culture for 48 hours was used. Hydrogen peroxide (H_2_O_2_; Sigma-Aldrich) was added to the culture medium at 50μM for 24 hrs. The nitric oxide (NO) donor, sodium nitroprusside (SNP; Baxter Healthcare Corporation, Deerfield, IL), was added to the culture medium at 0.5mM for 24 hrs. Vehicle treated cells served as controls. Cellular composition assay was performed 24 hrs after incubation at 37C°, 5% CO_2_.

### In vivo bioassay

Studies involving animals were performed with assistance of the DRI Preclinical Cell Processing and Translational Models Core under protocols (06–147) reviewed, approved and monitored by the University of Miami Institutional Animal Care and Use Committee (Animal Welfare Assurance A-3224-01 effective 12/4/02 with the Office of Laboratory Animal Welfare, National Institutes of Health). Female athymic nu/nu (nude) mice were obtained from Harlan Laboratories (Indianapolis, IN), housed in virus antibody–free rooms in isolated cages exposed to 12 hr light/dark cycle with *ad libitum* access to autoclaved/irradiated food and water at the Division of Veterinary Resources of the University of Miami. Diabetes was induced by streptozotocin injection and confirmed by detection of hyperglycemia (non fasting glycemic values >300 mg/dl on consecutive days). Under general anesthesia (inhalation of isoflurane/oxygen mix) mice received a 2,000 IEQ human islet graft under the kidney capsule, as previously described[8, 24]. Graft function was monitored by measuring nonfasting glycemic values (tail prick) with portable glucometers (Accu-Check, LifeScan). For assessment of cellular composition and antioxidant enzyme expression in different cell subsets, human islet grafts were excised 4 weeks after transplantation. Animals were humanely euthanized by exsanguination under general anesthesia (isoflurane, inhalation to effect).

### Islet dissociation and fixation

Aliquots of isolated islets were incubated for 10 min at 37°C using digestive enzyme (Accutase; Innovative Cell Technologies, San Diego, CA) and dissociated into single cells, followed by enzyme deactivation with cold fetal bovine serum. Single cell suspensions were mesh-filtrated and smeared on slide glass. After air dry, single cells were fixed with 2.5% paraformaldehyde (Electron Microscopy Sciences, Washington, PA) for 10 min at room temperature[25, 26].

### Immunohistochemical staining for detection of anti-oxidant enzymes

Tissue blocks were processed by the DRI Histopathology Laboratory. Human pancreata and islet graft were fixed in Bouin’s fixative solution (Sigma-Aldrich) for 6h. Human islets were fixed in Bouin’s fixative solution for 1h, dehydrated in 70% ethanol, and embedded in paraffin.

Sections (5μm-thick) were cut on a microtome, air dried overnight, deparaffinized, and rehydrated. After a wash (Optimax Wash Buffer, OWB; Bio-Genex, San Ramon, CA), sections and prepared slides were incubated with Universal Blocker Reagent (UBR; Biogenex),10% human serum for 10min, and washed with OWB. Thereafter, sections were incubated with rabbit anti-catalase (1:100, Rockland, Gilbertsville, PA), rabbit anti-SOD2 (1:100, Santa Cruz Biotechnology, Santa Cruz, CA), or anti-GPX (1:100, Abcam Inc, Cambridge, MA) antibody for 1hr. After washing, tyramide signal amplification (Dako, Carpinteri, CA) in combination with Alexa Fluor 488 antibody (1:200; Molecular Probes, Carlsbad, CA) was used. After staining, sections were incubated with mouse anti-C-peptide (1:100, Abcam Inc, Cambridge, MA), mouse anti-glucagon (1:500, Abcam Inc) for 2 hours at room temperature following incubation with goat anti-mouse Alexa Fluor 647 antibody (1:200, Molecular Probes) with DAPI for 1hr.

### Anti-oxidant enzyme expression on islet cells

Samples were analyzed with assistance of the DRI Imaging Core using Laser scan microscope (LSM) iCys (CompuCyte, Cambridge, MA) [8]. A minimum of 10,000 cells (events) was acquired and analyzed using mean fluorescence intensity (MFI) of each islet cell subset to compare the antioxidant expression, and define the percentage of antioxidant as below (mean MFI of antioxidant catalase, GPX, and MnSOD in positive population with islet subset marker positive population / mean MFI of antioxidant catalase, GPX, and MnSOD in whole population with islet subset marker positive population × 100).

### Assessment of cellular composition

Islet cellular composition was performed following the method previously reported[8, 27, 28]. Briefly, the slides prepared as mentioned above were utilized for this assay. After incubating with Protein Block and washing, cells were incubated for 2 hours with following antibodies: mouse anti-C peptide antibody (1:100), mouse monoclonal anti-glucagon antibody (1:500), and rabbit monoclonal anti-somatostatin antibody (1:500; Sigma-Aldrich). Staining samples were rinsed 3 times with OWB. Cells were incubated for 1 hour with either goat anti-mouse Alexa Fluor 488 antibody (1:200) or goat anti-rabbit Alexa Fluor 647 antibody (1:200). Omission of the primary antibody served as negative control. After washing, DAPI was applied to stain cell nuclei. Samples were then analyzed using LSC. A minimum of 5,000 cells (events) were acquired and analyzed for each sample. Cellular composition of each islet subset was compared.

### Necrosis assay

Necrosis assay was utilized after induction of oxidative stress to determine the viability of human β- and α-cells. Dissociated islets were incubated with 7-aminoactinomycin D (7-AAD; 10ug/ml; Molecular Probes) for 10 minutes. After washing 3 times with PBS, samples were fixed with 2.5% paraformaldehyde for 10 min at room temperature. Preparation was washed 3 times with OWB. After incubation in UBR, mouse anti C-peptide (1:100) or mouse anti glucagon (1:500) were added and for 30 minutes at room temperature. Thereafter, islet were washed with PBS 3 times and incubated with goat anti mouse Alexa Fluor 488 antibody (1:200) for 15 minutes.

Cell suspensions were analyzed (minimum 5.0x10^4^ events) using a LSR II flow cytometer with the FACSDiva^™^ software (Becton Dickinson, Mountain View, CA). The percentage of 7-AAD^+^ (necrotic) cells in C-peptide^+^ or glucagon^+^ population was calculated to determine the effect of oxidative stress inducing death of α- and β-cells.

### Assessment of DNA damage in islet cells

8-hydroxy-2′-deoxyguanosine (8-OHdG) is an oxidation byproduct of deoxyguanosine, a base in the DNA structure and as such its detection signifies DNA damage by oxidative stress. 8-OHdG is one of the predominant forms of free radical-induced oxidative lesions, and has therefore been widely used as a biomarker for oxidative stress and carcinogenesis. We investigated the extent of DNA damage from ROS in α- and β-cells by comparing the levels of 8OHdG following islet exposure to H_2_O_2_, as well as vehicle (control). H_2_O_2_ was added to the culture medium at 50μM for 24 hrs.

After human islet dissociation by Accutase, single cell suspensions were filtered, smeared on a glass slide, air-dried, fixed with 2.5% paraformaldehyde (Electron Microscopy Sciences, Washington, PA) for 10 min at room temperature[7]. After incubating with Protein Block, slides were incubated for 1 hour with mouse monoclonal anti-8-OHdG antibody (1:100, Abcam). Stained samples were washed with OWB 3 times. Signal expression was amplified using tyramide signal amplification in combination with Alexa Fluor 488. After washing, 4’,6-diamidino-2-phenylindole (DAPI) was applied to stain cell nuclei. Samples were analyzed using LSC/iCys[8, 27]. A minimum of 10,000 cells was acquired and analyzed for each sample.

### Microscopy and imaging

Stained sections were examined by the fluorescent microscopy at 20x and 40x magnifications. Fluorescent images were captured with a digital camera using Metamorph software (Molecular devices, Ontario, Canada).

### Statistical analysis

All statistical analyses were performed using R version 2.11 (http://www.R-project.org). Data are presented as means ± standard deviation (SD). Statistical difference between different groups were analyzed with Tukey-Kramer multiple comparison. For comparison of two groups, paired student t test was used. Findings are considered statistically significant if the corresponding P-value is <0.05.

## Results

### Diabetes alters human islet cellular composition and β/α-cell ratio

**Fig 1:**
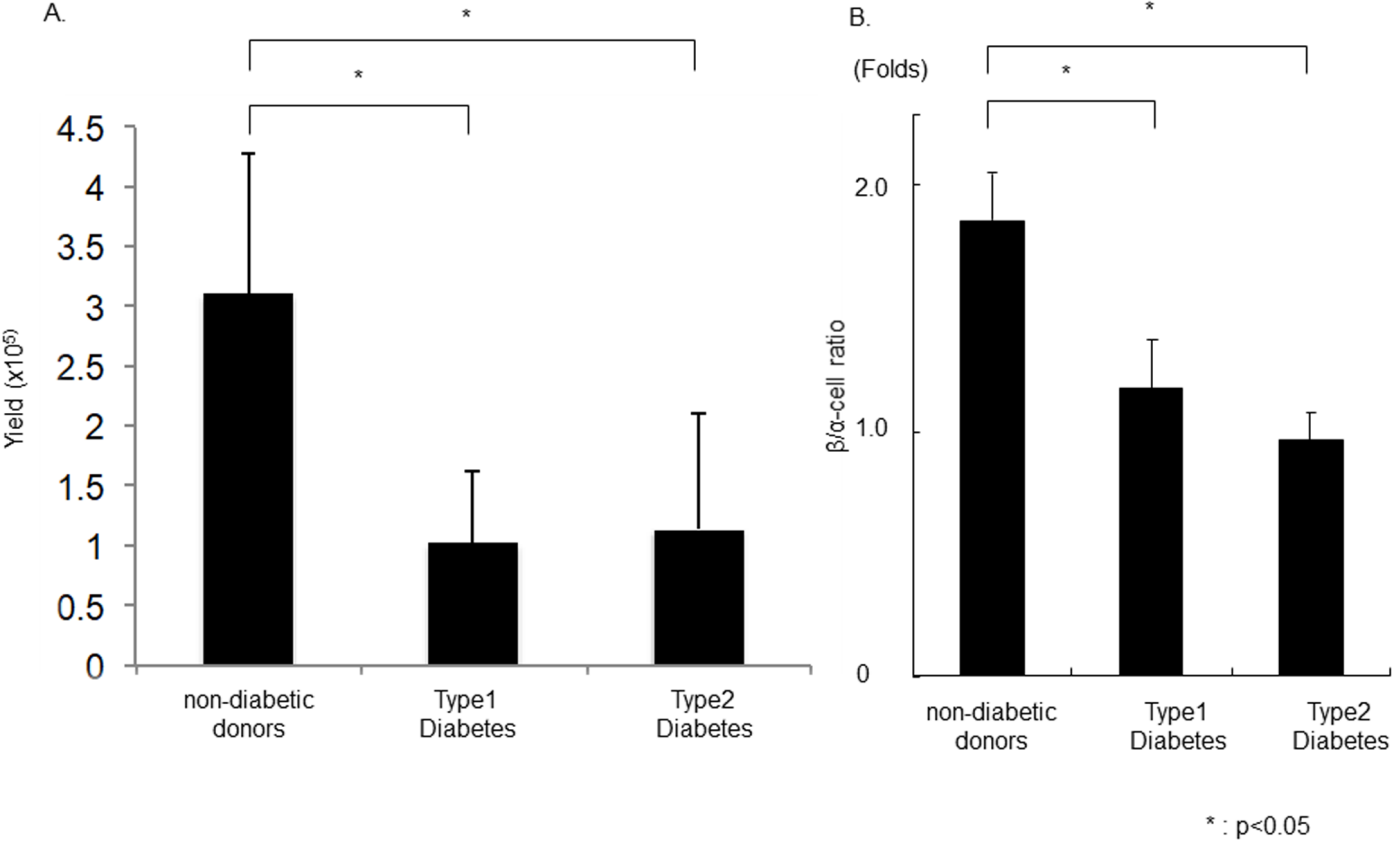
Cellular composition of islets from diabetic patients. (A)Human islet isolation from non-diabetes donor, type 1 or type 2 diabetes donors were performed. Islet isolation yield were significantly lower in diabetes donor than non-diabetes donor. (B)The cellular composition of isolated islets was evaluated and compared. The β/α-cell ratio was significantly lower in patients with Type 1 and Type 2 diabetes when compared with non-diabetic donors (N = 4).

Islet yields are generally lower from human pancreata obtained from patients with T1DM (1.02 ± 0.61 x 10^5^ IEQ) and T2DM (1.14 ± 0.96 x 10^5^ IEQ) than that of the non-diabetic donors (3.10 ± 1.17 x 10^5^IEQ) (Fig 1A). The β/α-cell ratio in T1DM and T2DM were significantly lower (1.24 ± 0.19 and 0.98 ± 0.17 Folds in T1DM and T2DM, respectively) than that of non-diabetic donor pancreata (1.88 ± 0.16 Folds; P<0.05; Fig 1B).

### Reduced β/α-cell ratio after human islet isolation and transplantation in the *in vivo* bioassay

**Fig 2:**
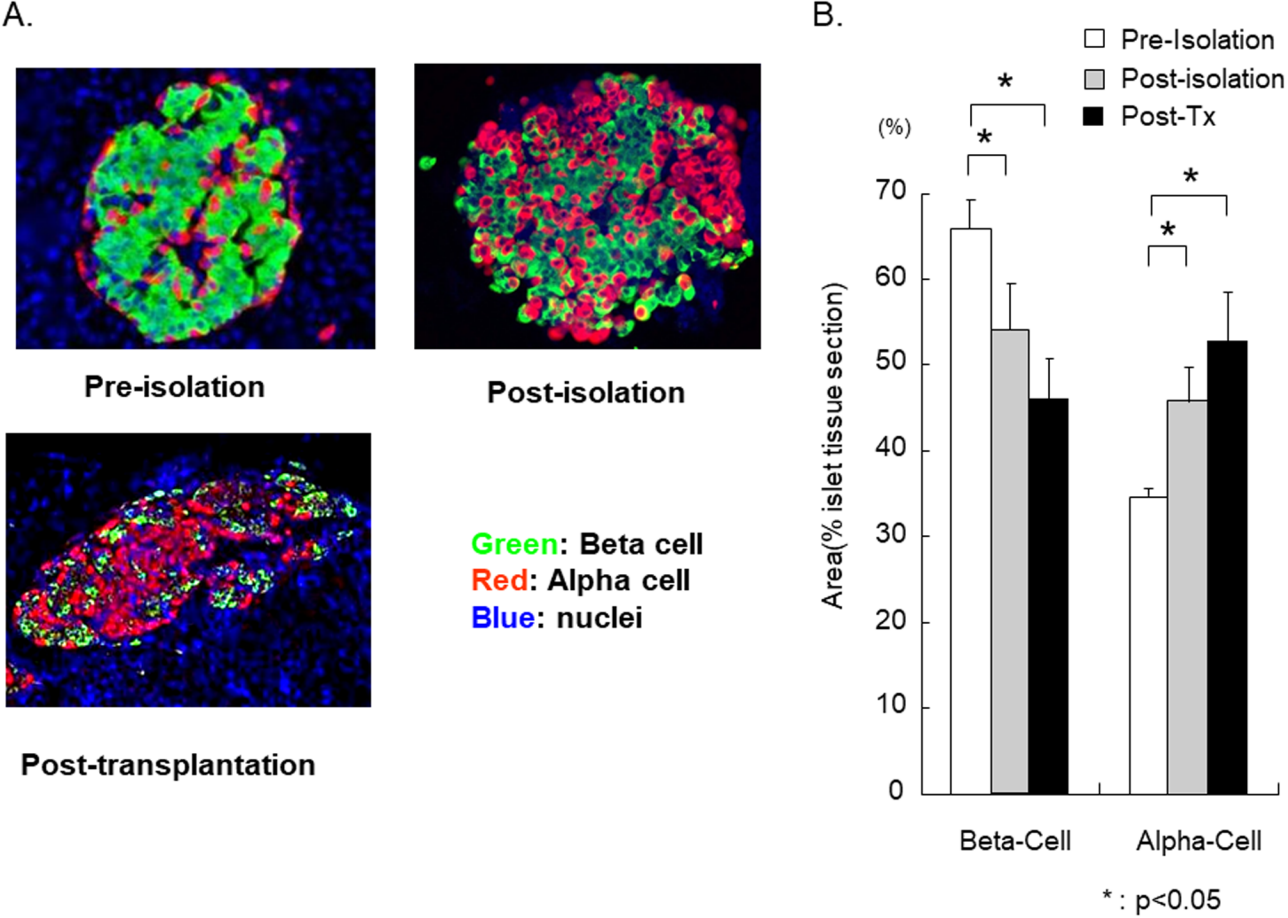
The β/α-cell ratio change pre-, post-isolation and post-transplantation. (A)Representative photograph of islet stricture. During islet isolation and transplantation, islets are exposed to mechanical, chemical, and hypoxic-reperfusion injury, generally leading to ROS generation. (B)The change of β/α-cell ratio in pre-, post-isolation, and post-transplantation into diabetic immune-deficient mice was examined. The β/α-cell ratio significantly decreased over the procedures, indicating a higher susceptibility of β-cells to the stress factors (N = 5).

The β/α-cell ratios in human pancreata from non-diabetic pancreas donors were determined before isolation (pancreas sections), after islet isolation, and after transplantation into diabetic immune-deficient mice. After islet isolation, area of β-cell significantly decreased from 65.4 ± 3.1% (pre-isolation) to 54.6 ± 6.0% (post-isolation; P<0.01). The area of β-cell post-transplantation was 47.6 ± 5.1%, which was significantly lower than the areas observed pre- or post- isolation (P<0.01; Fig 2A and 2B). On the other hand, area of α-cell significantly increased from 34.6 ± 1.1% (pre-isolation) to 45.4 ± 3.2% (post-isolation; P<0.01). The area of α-cell post-transplantation was 52.3 ± 7.4%, which was significantly higher than the areas observed pre- or post- isolation (P<0.01; Fig 2A and 2B). These observations suggest that human β-cells are more vulnerable to various insults occurring during the isolation and the early implantation period, including oxidative stress when compared to the α-cells.

### Differential expression of anti-oxidant enzymes in human β and α-cells

**Fig 3:**
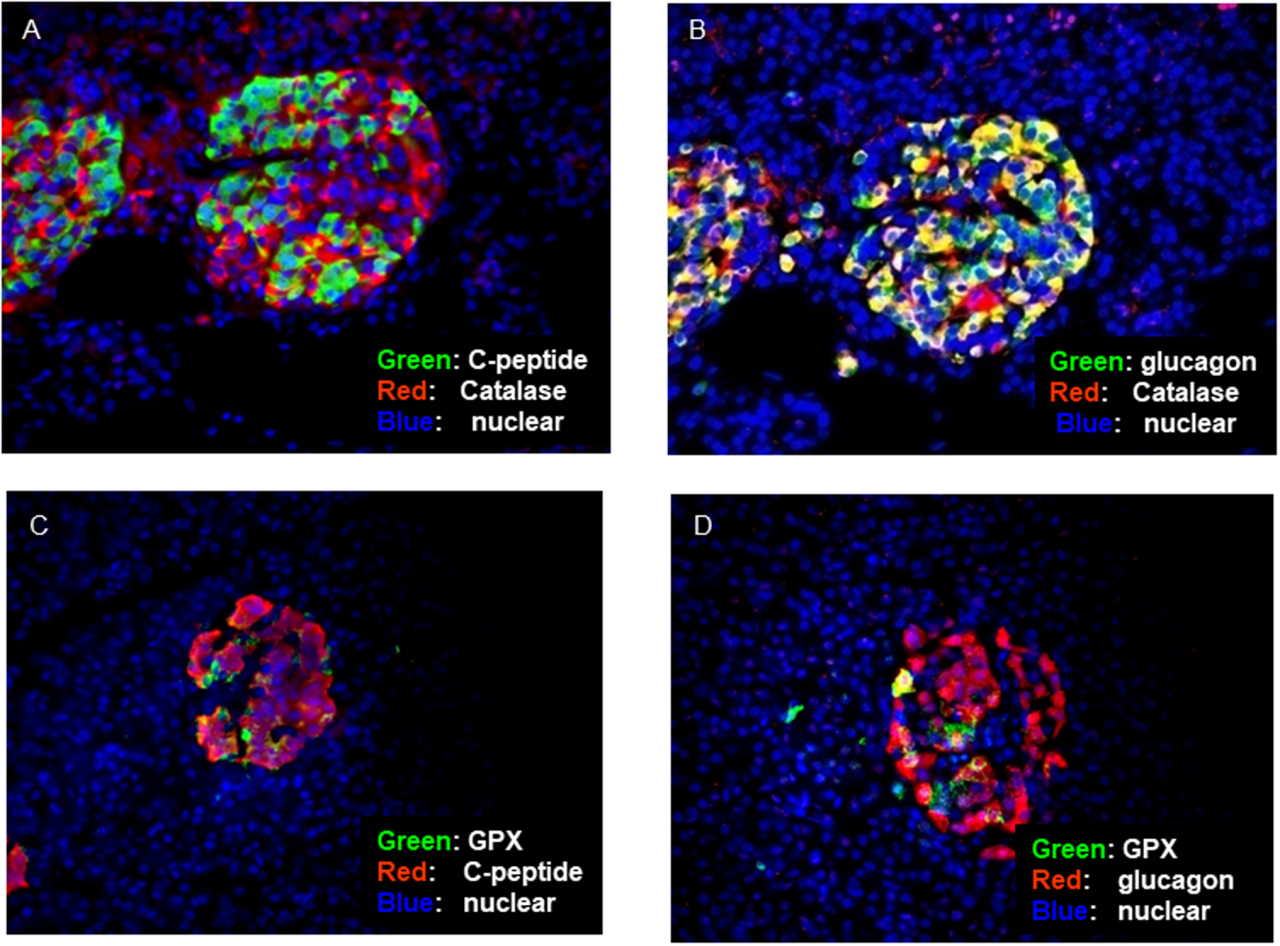
Anti-oxidant enzyme expression in non-diabetic human islet cell subset. (A and B) Anti-oxidative enzymes, catalase and glutathione peroxidase (GPX) expression of α and β-cells in non-diabetic human pancreas were examined using immunofluorescence. In representative picture, catalase was co-expressed with glucagon in a substantial proportion of cells and GPX was also found in some α-cells. (C and D)However, both enzymes were present only in very few β-cells(N = 5).

Catalase and GPX expression in human islets before isolation were examined to identify the anti-oxidant level in different islet cell subsets. Although limited number of β-cells expressed catalase, co-expression of catalase with α-cells was clearly confirmed (Fig 3A & 3B). In addition, co-expression of GPX with some α-cells was observed (Fig 3D).

**Fig 4:**
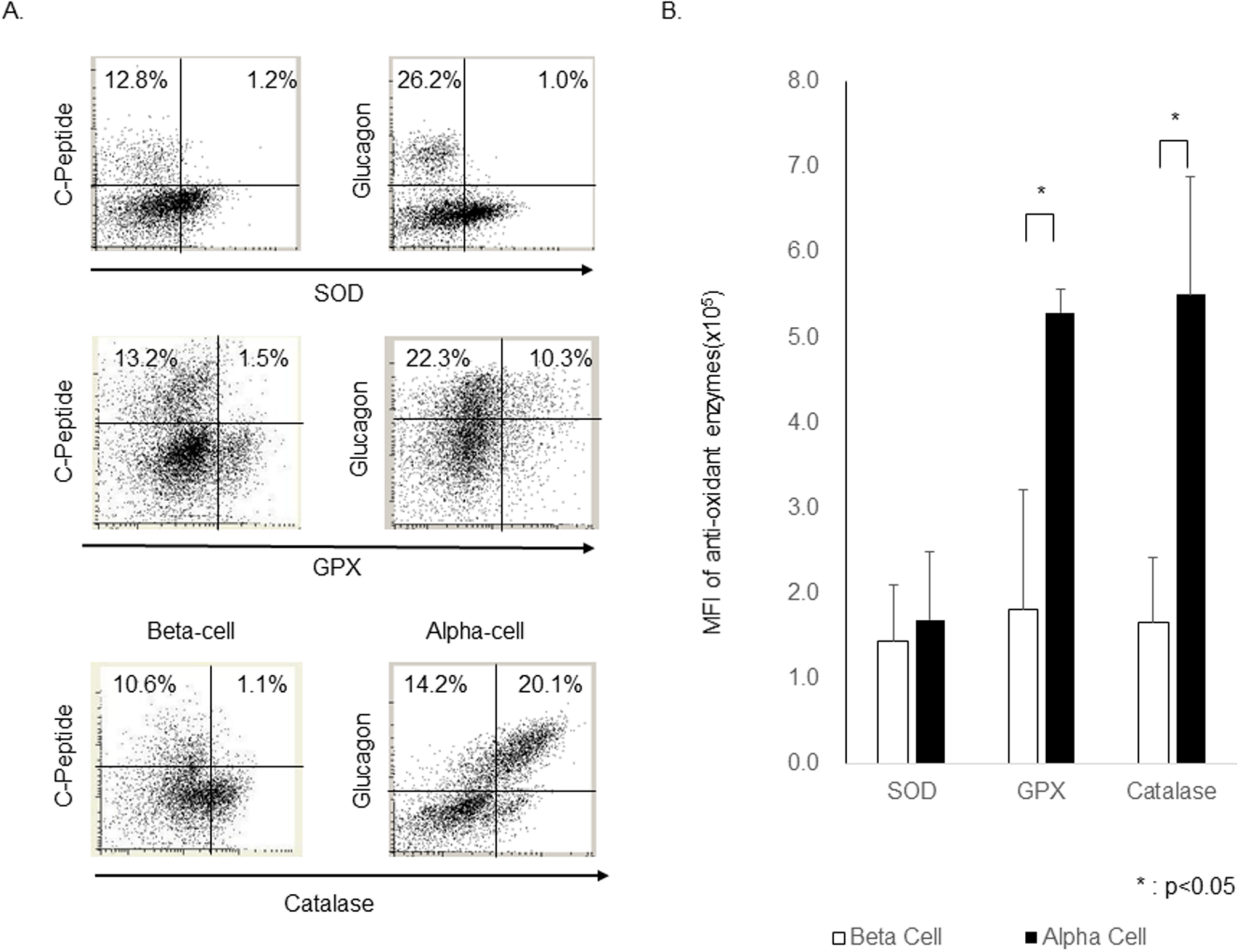
Quantification of Anti-oxidants expression in human islet cell subsets. (A) Representative plot of islet with expression of three anti-oxidant enzymes. (B) The level of MFI of three anti-oxidant enzymes in in human pancreatic α and β-cells was quantitatively evaluated using LSC/iCys. α-cell expression of catalase and GPX was significantly higher than that of β-cells. On the other hand, the difference of SOD expression in α and β-cell subsets was not observed. These data confirm the scarce expression of antioxidant enzymes in β-cells, which can contribute to their high susceptibility to ROS-mediated stress(N = 5).

To quantitatively investigate co-expression of anti-oxidant enzymes with each islet cell subset, stained islet cells were assessed by LSC/iCys. We confirmed that catalase was expressed in almost all α-cells and only in a small proportion of β-cells. In addition, GPX is expressed in many α-cells when compared to the β-cells. On the other hand, there was little SOD expression in both α- and β-cells (Fig 4A).

To compare the antioxidant expression level of each islet cell subset, the mean fluorescence intensity (MFI) of each islet cell was calculated, which demonstrated that catalase was highly expressed in α-cells (5.50 ± 1.53 x 10^5^) but the expression in β-cells (1.66 ± 0.74 x 10^5^) was limited. In addition, GPX expression in α-cells (5.29 ± 0.29 x 10^5^) was also significantly higher when compared to the β-cells (1.80 ± 1.51 x 10^5^). On the other hand, there was little SOD expression in both α- (1.69 ± 0.81 x 10^5^) and β-cells (1.45 ± 0.75 x 10^5^) (Fig 4B).

### Viability assay in β- and α-cells after exposure to oxidative stress *in vitro*

**Fig 5:**
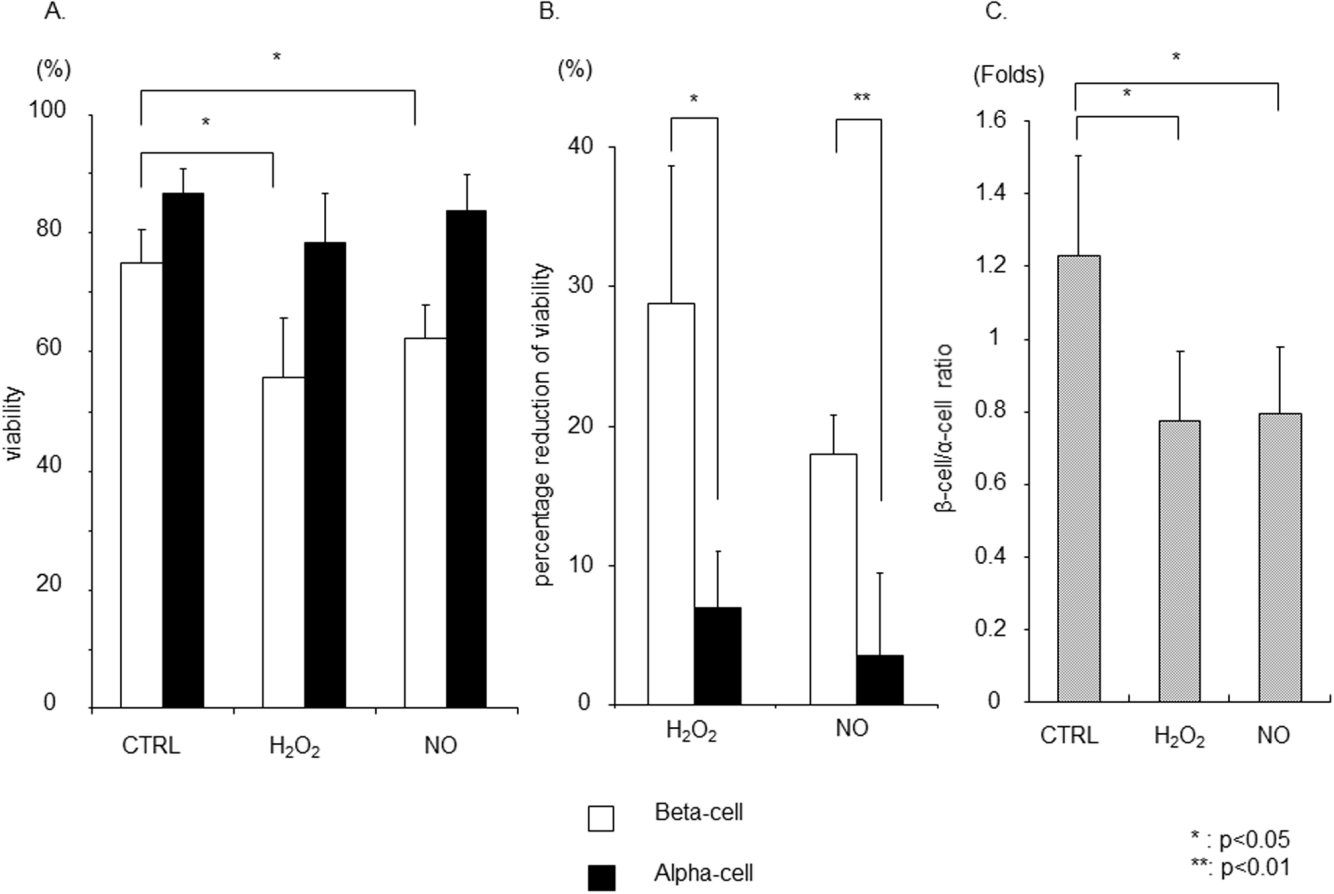
Viability assay in β-cells and α-cells and cellular composition change after oxidative stress. (A) Human islets were incubated with Hydrogen Peroxide (H_2_O_2_) or Nitric oxide (NO) for 24 hour. Pancreatic β-cell or α-cell specific necrosis was assessed using Flow cytometry. Although there was no significant difference in the viability of α-cells (7AAD negative population in glucagon positive population), β-cell viability significantly decreased. Reduction in viability was calculated as the difference of viability in the specific population in untreated control islets versus treated islets for each islet preparation. (B) The percentage reduction of viability in β-cell was significantly higher than that of α-cells. (C) The cellular composition in human islets after oxidative stress was assessed using LSC/iCys. The β-cell/α-cell ratio in islet cells after oxidative stress was significantly decreased(N = 5).

To test the hypothesis that β-cells are more vulnerable to oxidative stress due to the lower expression of anti-oxidant enzymes, human islets were incubated with H_2_O_2_ or NO donor for 24 hour. Pancreatic β- or α-cell-specific necrosis was assessed using flow cytometry. Although there was minimum change (CTRL: 88.3 ± 4.8%; H_2_O_2_: 79.6 ± 12.8% NO: 86.2 ± 8.4%; Fig 5A) (P = NS) in the viability of α-cells (7AAD^−^ population in glucagon^+^ cells) after treatment of H_2_O_2_ or NO, β-cell viability was significantly decreased (CTRL: 76.1 ± 8.2%; H_2_O_2_: 57.3 ± 11.3%, P<0.05; NO: 61.9 ± 6.2%, P<0.05; Fig 5A). The reduction in viable β-cells following exposure to H_2_O_2_ or NO donor was significantly higher than that observed in α-cells (H_2_O_2_: 28.7 ± 9.9% vs. 6.9 ± 4.1%, P<0.05; NO: 17.9 ± 3.0% vs. 3.5 ± 6.0%, P<0.01; Fig 5B). The cellular composition of human islets exposed to oxidative stress *in vitro* was also assessed using LSC/iCys. The β/α-cell ratio significantly decreased in islet cells following exposure to H_2_O_2_ or NO. Taken together, the result clearly demonstrated that β-cells are more vulnerable to oxidative stress than α-cells (Fig 5C).

### Human β-cells is more susceptible to oxidative stress-mediated DNA damage

**Fig 6:**
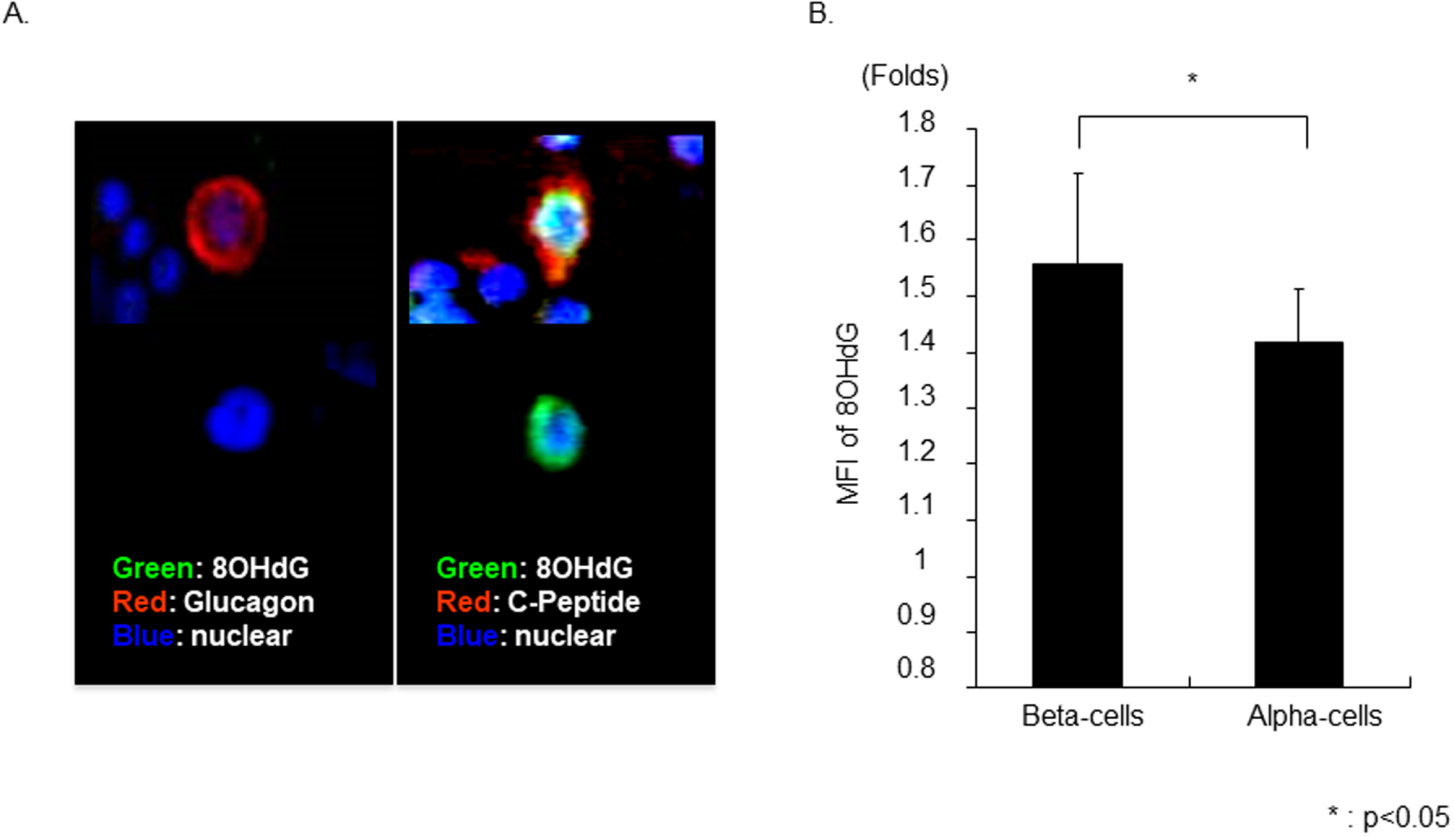
8-hydroxy-2’-deoxyguanosine expression in α- and β-cell after oxidative stress. (A) Representative photograph of α- and β-cell with or without expression of 8OHdG after giving oxidative Stress. 8OHdG expression in β- or α-cells was assessed using LSC/iCys. (B) The average MFI level of 8OHdG in α-or β- cell subset after exposure to H_2_O_2_ were calculated and compared to the vehicle (non-stimulated group). The amount of 8OHdG after oxidative stress significantly increased in β-cell, when compared to α-cell (N = 5).

To confirm oxidative stress-induced DNA damage in β- and α-cells, 8OHdG intensity was assessed by LSC/iCys. The average MFI level of 8OHdG in α-or β- cell subset after exposure to H_2_O_2_ were calculated and compared to the vehicle (non-stimulated group). The increase in 8OHdG level was significantly greater in the β-cells compared to α-cells (1.56 ± 0.16 vs. 1.41 ± 0.09 Folds; P<0.05; Fig 6). These results demonstrated that oxidative stress significantly increased DNA damage in β-cells when compared to α-cells.

## Discussion

The key findings of the present study include: (i) Reduction of β/α-cell ratio in human islets from patients with T1DM and T2DM; (ii) consistent decrease in β/α-cell ratio during islet isolation and following islet transplantation; (iii) greater vulnerability of β-cell than α-cell to oxidative stress; and (iv) lower expression of the key antioxidant enzymes catalase and GPX in human β-cells than α-cells, a phenomenon which could, at least in part, account for the higher oxidative damage and lower viability of β-cell following exposure to oxidative and nitrosative stress observed.

Selective destruction of pancreatic β-cells plays a central role in the pathogenesis of both T1DM and T2DM. In T1DM, the infiltration of the pancreatic islets by immune cells mediates β-cell dysfunction and destruction by production of pro-inflammatory cytokines and reactive oxygen and nitrogen species [8, 27, 28] resulting in insulin deficiency. T2DM is characterized by abnormal insulin secretion, associated with varying degrees of insulin resistance, caused by a combination of genetic and acquired factors that impair β-cell function and tissue insulin sensitivity[29, 30].

β-cell dysfunction is central to the development of T2DM and is likely due to a combination of decreased β-cell mass and defective insulin secretion. Several studies have demonstrated the association of T2DM with increased β-cell apoptosis, impaired β-cell replication/neogenesis and reduced functional β-cell mass[31, 32]. Glucose toxicity and lipotoxicity are among factors that have been investigated as mediators of β-cell damage in T2DM[33–37]. The theory of glucose toxicity proposes that continuous exposure to modest increases in blood glucose over a long period could adversely affect islets by promoting generation of ROS[38–41]. Several mechanisms have been proposed to explain the deleterious effects of prolonged exposure to high glucose on islet cells, including increased oxidative phosphorylation, over-activation of the hexosamine pathway, and enhanced activity of protein kinase C, among others[42, 43]. Notably, all these pathways lead to augmented production of ROS. Lower level of the antioxidant enzymes in β-cells can explain their increased vulnerability to oxidative stress, and contribute to the observed lower β/α-cell ratio in T2DM in our study.

ROS play a critical role in causing cellular and tissue damage and dysfunction[38–40, 44]. In fact confirming the results of the present study several reports have indicated that pancreatic islets are particularly vulnerable to ROS such as H_2_O_2_ and superoxide[12–19].

A small but significant portion (1–4%) of oxygen processed in the mitochondria is converted to ROS. The primary ROS produced in mitochondria are superoxide anion (O2•-) and H_2_O_2_. Generation of ROS in mitochondria increases in response to hypoxia, ischemia, elevation of substrate such as glucose, or their combination. In addition to mitochondria, a number of cytosolic enzymes, including oxygenases, oxidases, and peroxidases, generate superoxide and hydrogen peroxide. Although excess generation of hydrogen peroxide and superoxide can cause oxidative stress and cytotoxicity, when produced at a normal rate, they are safely neutralized. For instance, under normal condition superoxide is converted to H_2_O_2_ by SOD isoforms, and H_2_O_2_ is converted to water by catalase and glutathione peroxidase. However, in pathological conditions, they can serve as substrates for the generation of highly reactive and cytotoxic products such as hydroxyl radical (•OH), peroxynitrite (.ONOO), and hypochlorous acid (HOCl). Moreover, during the course of inflammation, activated leukocytes, macrophages, and resident cells generate considerable amounts of ROS and reactive nitrogen and halogen species. These highly reactive and cytotoxic molecules mediate tissue damage and dysfunction by inducing necrosis, apoptosis, inflammation, fibrosis, and DNA damage[45]. Thus, oxidative stress occurs as a result of either increased ROS production and/or impaired antioxidant defense system. There is evidence that pancreatic islets are more susceptible to the ROS-mediated injury than other tissues. This is, in part, due to the low level of antioxidant enzymes in β-cells[41–43]. In fact, overexpression of antioxidant enzymes has been shown to protect rodent β-cells against oxidative stress [16, 46] and administration of ROS scavengers or antioxidant enzyme mimetic compounds [18, 47] has been shown to protect islets from ROS-induced injury in animal models of diabetes and islet transplantation. The susceptibility of β-cells to oxidative stress is compounded by presence of hyperglycemia that by increasing the glucose load of the mitochondria in β-cells heightens their intracellular ROS production. The associated oxidative stress provokes inflammation that can work in concert to cause β-cell death and dysfunction[17, 48–50].

Although the susceptibility of pancreatic islets to oxidative stress and its role in the pathogenesis of diabetes has been demonstrated, due to the structural complexity of the pancreatic islets, quantitative comparison of antioxidant enzymes in the islet cell subsets has not been reported previously. We quantified the expression of the key antioxidant enzymes using LSC/iCys that divides fluorescent signals representing the cell type and the target anti-oxidant enzyme on the two-dimensional graph. The quantity of catalase and GPX in β-cells was much lower than α-cells. Our data corroborate and extend previous knowledge that β-cells have poor antioxidant pool. We corroborate that significant differences in catalase and GPX expression exist between islet cell subsets, which might account for the fact that vulnerability and hence survivability of β- and α-cells to ROS may be different. This was confirmed by revealing that human islet exposure to H_2_O_2_ or NO donor resulted in significantly greater necrosis and reduced viability in β-cells than in the α-cells.

Moreover, DNA damage from oxidative stress, measured with 8-OH-dG, which is the byproduct of the DNA base modification by ROS [51], was significantly greater in the β-cells than in α-cells following exposure of human islets to H_2_O_2_ or NO donor *in vitro*. Therapeutic approach against ROS with reagent or cell therapy may save islets from islet cell death [52, 53].

## Conclusion

The present study revealed that expression of key antioxidant enzymes, catalase and GPX is much lower in human β-cells than in the α-cells. This was associated with significantly lower survival and viability and higher DNA damage in β-cells than α-cells following exposure to oxidative stress. These findings suggest that the lower β/α-cell ratio observed in human islets from patients with T1DM and T2DM, as well as the consistent fall in β/α-cell ratio following islet isolation and transplantation process could be in part due to the high vulnerability of β-cells to oxidative stress. Potential strategies aimed at enhancing β-cell antioxidant defense capacity might be effective in prevention/treatment of diabetes and improvement in the islet transplant outcome.
